# A Bayesian Target Predictor Method based on Molecular Pairing Energies estimation

**DOI:** 10.1038/srep43738

**Published:** 2017-03-06

**Authors:** Antoni Oliver, Vincent Canals, Josep L. Rosselló

**Affiliations:** 1 Physics Department, Universitat de les Illes Balears, Palma de Mallorca, Spain.

## Abstract

Virtual screening (VS) is applied in the early drug discovery phases for the quick inspection of huge molecular databases to identify those compounds that most likely bind to a given drug target. In this context, there is the necessity of the use of compact molecular models for database screening and precise target prediction in reasonable times. In this work we present a new compact energy-based model that is tested for its application to Virtual Screening and target prediction. The model can be used to quickly identify active compounds in huge databases based on the estimation of the molecule’s pairing energies. The greatest molecular polar regions along with its geometrical distribution are considered by using a short set of smart energy vectors. The model is tested using similarity searches within the Directory of Useful Decoys (DUD) database. The results obtained are considerably better than previously published models. As a Target prediction methodology we propose the use of a Bayesian Classifier that uses a combination of different active compounds to build an energy-dependent probability distribution function for each target.

Virtual screening (VS)^1^ is the automatized inspection of molecular libraries to identify those molecules that most likely bind to a given drug target. In molecular docking, the process of molecular binding to a biological target (normally a protein) is simulated to estimate the binding energy and therefore the likelihood of the molecule of being active. The main drawbacks of molecular docking are the precision in the prediction of the binding affinity^2^ and the high computational cost. The complexity to make predictions about binding interactions is due to different reasons as the flexibility of the protein and the ligand, the presence of water molecules in the crystal X-Ray structures or the existence of more than one active interaction site for the same target. Additionally, molecular docking implies the use of huge computational resources. For example, for simulating the docking of one million ligands the VinaLC software need the order of 1.4 hours by using 15,000 CPU cores^3^.

Ligand-based methodologies^4^ are frequently applied when the precise information about the 3D structure of the biological target of interest is lacking. This technique consists in the research for compounds that most closely resemble a given query molecule with known biological activity. The assumption is that similar molecules are likely to share similar properties. The similarity can be related to geometrical descriptors or to more elaborated chemical parameters.

One of the most widely used descriptors is binary fingerprint that describes molecular substructures by using a Boolean description. Fingerprints incorporate different information as molecular descriptors^5^ such as structural fragments^6^, possible connectivity pathways through a molecule^7^ or different types of pharmacophores^8^. They are also used by similarity search engines^9^, or in VS processes^10^. Nevertheless, the majority of fingerprint models do not include three-dimensional information and therefore conformational dependence is not considered. Other ligand-based methodologies are based on the use of different molecular characteristics as the shape^11^ or the charge distribution^12^. The main advantage of those similarity-search methods is the possibility to screen huge molecular databases in reasonable times. Their associated libraries can be composed by billions of compounds, thus improving the quality of their results.

The similarity search method is also an exceptional tool to identify off-target interactions. The estimation of cross-interactions between known compounds and drug candidates is of vital importance when considering the possible adverse drug reactions (ADR) that cross-interactions may cause (that would provide the possible side effects of the drug candidates). In fact, it has been reported that nearly the 35% of the drugs present in the market may have interactions with at least two different receptors^13^ that may lead to unwanted adverse effects.

A widely used Ultra-fast screening methodology was developed by Pedro Ballester *et al*.^11^ (the USR method). Due to the use of a small set of parameters (a total of 12 molecular descriptors), the model is able to achieve fast VS speeds. Since the geometrical approach is conformation dependent, a more realistic study can be done if a multi-conformational database is screened. The method is geometric so the chemical composition is not considered. The USR method is a particularly fast technique that can be applied to screen huge databases^14^ and its application^15^ has resulted in the discovery of molecules with previously unknown activity against a range of molecular and cell targets^16^,^17^,^18^,^19^.

Different models have been developed improving the USR method by including chemical parameters. USRCAT^20^ is an extension of USR including pharmacophoric information whilst retaining the performance of the original model. Other method based on USR is Electroshape^12^, that incorporates the molecular charge distribution. As a result, the new method increases the number of descriptors from 12 to 15, thus decreasing the screening speed by a 25%. Other recently published geometrical model is SHeMS^21^, that is based on the use of spherical harmonic expansions but do not consider the molecular charge distribution.

Other model that considers both the geometry and charge distribution is Mol-ShaCS^22^. This model use Gaussian descriptors for charge and volume and provides acceptable averaged ROC (Receiver Operating Characteristic) scores. The main drawback is the low processing speed (with up to 21 compared compounds per second as reported in ref. 22).

In the present work we propose a new molecular model based on the use of energy descriptors. The model is based on the estimation of the atomic partial charges. In this paper we use different ways to obtain those charges such as Gasteiger-Marsili^23^, Merck Molecular Force Field (MMFF94)^24^ and charge transfer polarization and equilibration (QTPIE)^25^. The methodology finally used is MMFF94 that provides the best results in different studies^26^,^27^.

To test the proposed model we use the Directory of Useful Decoys (DUD)^28^. The DUD database is related to a total of 40 protein targets, where a set of active compounds and decoys (that are presumed to be inactive but with similar physical properties with respect to active compounds) must be differentiated. The mean number of decoys per active is 36, and the total database size is of the order of 100.000 molecules. The set of DUD compounds is further discussed in ref. 29.

For the validation of the model we use enrichment curves in which the true positive and the false positive rates are represented in a graph. From these curves, two parameters are estimated such as the Area Under the Curve (AUC) and the Enrichment Factor at the 1% of the ranked database (EF_1%_). The enrichment factor provides information about the number of times in which more ligands are found with the method than would be expected if compared to a random picking and the AUC estimate the goodness of the proposed ranked compounds in the full database and not only in the first fraction of the ranking (as the EF parameter does). There is a vast number of methods present in the literature using those ROC parameters with the DUD database as a reference^11^,^12^,^22^,^30^.

One of the main disadvantages of similarity search models is that a single molecule is not enough to cover the whole range of compounds that can bind to a particular target receptor. Therefore, more elaborated methodologies considering different active compounds at the same time are needed. The diversity of the target binding sites can be considered by using a Bayesian methodology in which a multi-target predictor can be implemented. The Bayesian classifiers are based on the estimation of both the a priori probability and the probability density function for each measurement (the likelihood function) in order to obtain the a posteriori probability (that is the probability of the query molecule to be active against a given target). For the estimation of the likelihood function we use the Parzen window method that was developed by Emanuel Parzen^31^ and Murray Rosenblatt^32^ independently in the early 1960s. It has been used in a wide range of pattern recognition applications^33^,^34^,^35^ and has been enhanced in refs 36, 37, 38. The performance of the target predictor has been tested by using both the DUD and the ZINC databases.

## Energy descriptors

In this document we present a compact chemical model for an efficient characterization of molecular compounds where the information of the compounds’ pairing energies is used. At each atom position the partial charges are estimated using the MMFF94 method that is implemented within the Openbabel software. For every pair of atoms in the molecule (labeled as “i” and “j”) with partial charges q_i_ and q_j_ and Euclidian distance r_ij_ between them, the pairing energies are defined as follows:


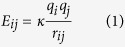


where “κ” is the Coulomb energy constant (κ = 14.4 eV Å/e^2^). For a compound containing a total of ‘N’ atoms the number of pairing energies is:


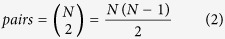


**Figure 1:**
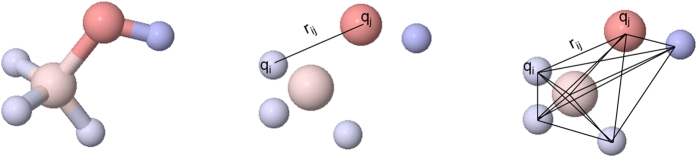
Estimation of the energy descriptors associated to the methanol. The original molecule (left) is reduced as a set of discrete atom points (middle). A total of fifteen pairing energies can be estimated from the resulting distribution.

The method is shown graphically at Fig. 1 for the characterization of methanol.

**Figure 2:**
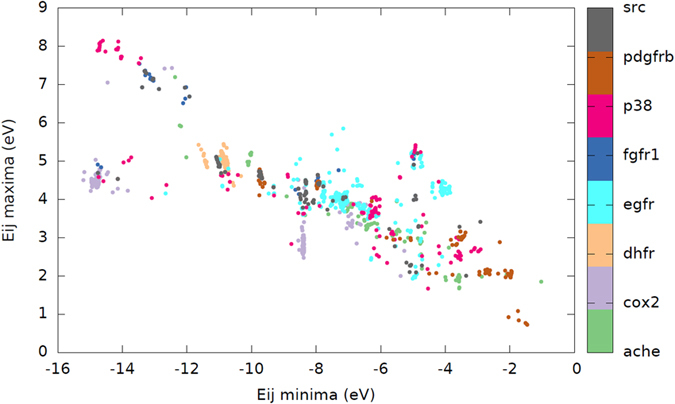
Two-dimensional pairing-energy map for eight DUD targets. The energy values shown are the most positive and negative pairing energies. As can be appreciated, different clusters are associated to each specific targets.

A minimum threshold distance is defined in order to filter adjacent charges closer than 1.0 Å. The pairing energies can be understood as being those physical parameters providing the information about the local binding energies between each atom pair independently of the rest of the molecule. Pairing energies are therefore local and are more prominent when two high-polarity atoms are very close (with a low r_ij_ value). The pairing energies present a clear clustering effect depending on the type of compound and the specific therapeutic target with respect is active, therefore they are suitable to be used as molecular descriptors. In Fig. 2 we show a two-dimensional energy map for the active compounds of eight different targets taken from the DUD database. As can be appreciated, a clustering effect is observed even using two dimensions (with the most positive and most negative pairing energies). Each target can produce different clusters that can be explained as being associated to different binding sites of the therapeutic target.

The proposed descriptors for the molecular characterization are obtained by estimating all the pairing energies. These values are ranked so that both the m/2 most positive and negative energies are selected as description set. As a result, an m-dimensional energy vector associated to each compound is created. The selected pairing energies provide information of both the geometry and charge distribution for the most polar sections of the molecule (closer molecular regions with higher charge values). Therefore, the selected pairing energies associated to a given compound (to create the energy vector) will be a representative identification of the most active molecular regions.

The Pairing Energy Description model (PED) presented in this work uses an energy vector **E** that can be used for virtual screening, clustering or to estimate the compound’s most probable targets. In this work we compare the proposed methodology with previously published models^11^,^12^,^22^ by using the DUD database. A similarity metric is used to energetically compare two molecules (and therefore assume a similarity in their chemical activity):


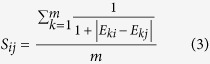


Using parameter S_ij_, the most similar compounds can be identified, where a S_ij_ value close to 1 reveals compounds with similar binding properties. Parameter ‘m’ is the number of pairing energies used in the descriptor vector (in this work we used three different values for this parameter that are 2, 6 and 12). The application of (3) in the selection and classification of the possible active molecules for a given target is presented in the results section.

## A Bayesian classifier for Target prediction

To infer which possible Target can be associated to a given compound we need to estimate the a-posteriori conditional probability P(C_k_|**E**) that is defined as the probability of a given molecule to be related to target ‘k’ given an energy vector **E** is measured. For this purpose we first have to estimate the a-priori probability P(C_k_) and the likelihood function P(**E**|C_k_) that is the probability distribution function of all the active compounds that are associated to a given target C_k_. Using the active set of drugs associated to target C_k_ we can construct the likelihood function by using the Parzen-windows approximation:


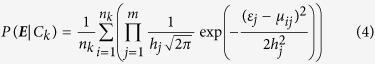


where vector **E** is defined as **E** = (ε_1_, ε_2_, ..., ε_m_), n_k_ is the number of kernels presenting activity with respect to the target ‘k’ (number of active compounds considered for the construction of the likelihood function), and μ_ij_ is the j-th pairing energy value of the i-th kernel that has been selected. Parameter h_j_ is the window bandwidth for each dimension that has been found to be optimal following the next expression:


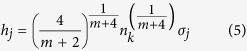


where σ_j_ is the standard deviation of the distribution of all the j-th component of the kernel’s energy vectors. Using the Likelihood function provided by (4), the probability of obtaining the class C_k_ given that **E** is measured P(C_k_|**E**) can be estimated by applying the Bayes theorem (6) and assuming that the a priori probabilities of each target class is P(C_k_) = n_k_/N, where N is the total number of possible compounds and n_k_ the number of actives belonging to the ‘k-th’ class. Then, for M classes (representing different targets), the next expression provide the probability of a compound to belong to class C_k_ given that vector **E** has been associated to this compound:


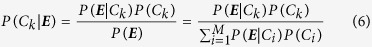


## Results

### Qualitative analysis

In order to check the capacity of the method to select molecules with similar polarities, three query molecules have been retrieved against ZINC all purchasable dataset using expression (3) to estimate the similarity. The molecules have been previously filtered using a similarity threshold value (S_ij_ > 0.9), and then ranked with respect the similarity score value. Finally, the top ten molecules are finally selected and shown in Table 1. At the Supplementary information, the graphs of these top ranked compounds are presented with the ZINC codes.

The query molecules selected are Favipiravir, Ibuprofen and Caffeine. As is shown at the Supplementary Information, the proposed method preserves the active electrostatic regions of the query molecule while non-polar regions are not necessary maintained. The partial charges play an important role in this effect. For the case of Favipiravir, exactly the same polar region has been found on the first hits, only changing the halogen atom, which is Fluorine in Favipavir (ZINC13915654 code), Bromine for ZINC33961890 and Chlorine for ZINC88190053. Other similar compounds present hydrocarbon tails and low energy functional groups added to the main polar region. The same observation is valid for Ibuprofen and Caffeine queries.

### ROC evaluation

**Figure 3:**
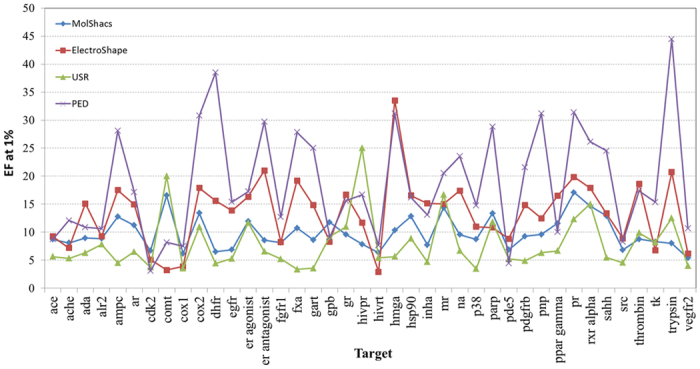
Comparison of EF at 1% between the USR (green line), ElectroShape (red line), MolShacs (blue line) and the proposed model PED (purple line) with m = 12 and MMFF94 partial charges for ElectroShape and PED.

The validation of the proposed model has been done using the DUD database in different ways. Firstly, a simple similarity search for all active compounds for a given target is done against the rest of the database, in this way the ROC curve is built (True positive vs. false positive curve). The comparison is made using the metric shown in (3), and finally the compounds are ranked by energy similarity. Then, a mean ROC curve is calculated by averaging all the curves provided by all active compounds. The Area Under the Curve (AUC) and the Enrichment Factor at 1.0% are then calculated. The EF_1%_ values is shown at Fig. 3 for each target when m = 12. The proposed model (labeled as PED), is compared with USR^11^, MolShacs^22^ and ElectroShape^12^.

As can be appreciated in Fig. 3, for targets pnp, dhfr, hmga or trypsin, the proposed model presents better results in comparison with other models (USR, ElectroShape and MolShacs). On the other hand, PED is not presenting the better EF value for targets comt, pde5, cdk2, ppar gamma and gpb.

Normally, targets with a small number of compounds provide ow EF values (for example, the target hivpr only have 4 actives). For a wider comparison, in Table 2 we show the mean and the standard deviation of the results of the proposed model with respect to other models present in the literature^11^,^12^,^22^,^30^. We use different dimensions for the proposed model (m = 2, 6 and 12) as a reference. The results show that ElectroShape presents the best AUC values while that the PED model has better enrichment factors. The EF values are more relevant than AUC metric when considering Virtual Screening of huge databases (with millions of compounds) in which only a small percentage (the top ranked one) will be posteriorly tested in the laboratory (the AUC metric is extended to the whole ranking). Therefore, the AUC metric is not very suitable to provide information about the Virtual Screening performance of the models^39^. In addition, the standard deviation of the EF values present a large variability between categories, as it can be observed from Fig. 3. At the same time the use of a low number of descriptors implies less memory resources used and a higher processing speed. Considering that ElectroShape use a total of 15 descriptors, then the proposed model is 2.5 or 7.5 times faster when using m = 6 or 2 respectively.

### Application to Massive Virtual Screening

The results obtained using the DUD database indicate that the proposed model can differentiate actives from decoys with a relative good successful rate. To check if the method works in a more realistic experiment, a massive virtual screening experiment is done. For 10 random and active compounds taken from the DUD database, a massive search against the full DUD database and the entire subset of purchasable compounds of ZINC database (around 20 million of compounds) has been implemented. The idea of this experiment is to find the number of compounds with the same activity of the query in the first parts per million (ppm) of the database (top 20, top 10 and top 5). Previous to the experiment, the query molecule is removed from the database. The results of the experiment are exposed in Table 3.

The results show that the compound’s pairing energies can be used to find more compounds with biological activity in large databases if compared with previously published models. One of the main advantages of the presented descriptors is the fast processing speed achieved due to the simple calculations that are involved. For instance the pairing energies can be calculated with a speed close to 100 compounds per second using a single processor on i7-Core laptop. Therefore, the creation of large datasets is feasible. On the other hand, the speed of comparisons using expression (3) is very similar as the obtained using USR descriptors^11^.

### Bayesian application using the Parzen-window density estimation

**Figure 4:**
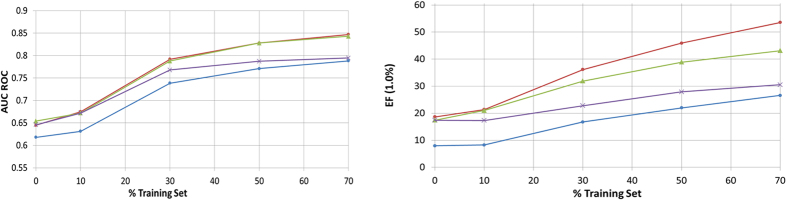
AUC and EF at 1% for the proposed model (red for m = 12, green for m = 6 and purple for m = 2) and USR (blue line) models. Averages for different fractions of the training set are estimated (10%, 30%, 50% and 70% of the actives). The origin represents the averaged performance of single similarity retrieving showed at Table 2.

Here we present the extension of the model using the Bayesian estimator (4), with the same role as expression (3) but considering the capacity of joining different active compounds in each search at the same time. In our study, we randomly selected a given percentage of the actives provided by the DUD database (10%, 30%, 50% and 70%) to be included in the Bayesian classifier (we define those selected actives as the Bayesian kernels). The rest of the database (that is, a 90%, 70%, 50% and finally a 30%) is included inside a Test set (that also is containing all the DUD decoys). Since the results are sensible to the specific molecules selected to be included in the Bayesian kernel, a total number of 100 different kernels (or training sets) have been selected to reproduce the associated ROC curves (that are posteriorly averaged). The results of these experiments (the AUC and EF values of the success of the target prediction in the test set) are shown in Fig. 4. As can be appreciated, the AUC and EF values increases considerably as the training set (included as kernels in the Bayesian classifier) is increased. In the experiments we also changed the vector dimension from m = 2 to m = 12. As a comparison, we also implement a Bayesian classifier to the USR model.

As expected, the results show a clear dependence of the AUC and EF_1.0%_ success with the training set size (number of compounds included as kernels in the likelihood function). In the case of the Enrichment Factor we observe that the improvements obtained as the training set size is increased are greater for the proposed descriptors than the USR set. For the case of m = 2 and 6, the results are still acceptable and also better than USR.

### Threshold estimation

A target prediction application has been build using a database with different target proteins and actives. For all the targets, a probability threshold (P_*TH*_) is defined in the intersection of the ranked positive rate (i.e. actives that are correctly selected) and the negative rate (i.e. decoys that are wrongly selected). The target predictor use expression (7), in which the estimated probability is compared with the threshold value ‘P_*TH*_’ that have been selected.


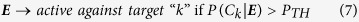


For the estimation of the optimum threshold we develop a target predictor application that has been tested by comparing each active compound (test) against all the DUD actives (the tests compounds have been previously erased from the training set). Then, all target classes have been represented by a 12-dimensional a-posteriori probability that is composed by all active compounds excluding those belonging to the test set. For simplicity the molecules with more than a single activity in the DUD database have been removed, remaining only 2.048 compounds. After that, the probability of each compound to belong to a given class has been calculated using (6).

**Figure 5:**
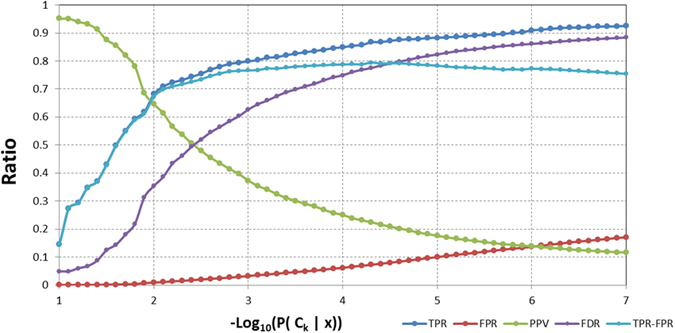
Logarithmic plot of the probability histogram for TPR (blue) and FPR (red) and the difference between them TPR-FPR (light blue). The Positive Predictive Value (PPV, i.e. precision, in green), and the False Discovery Rate (FDR, purple) are shown in the same graph.

Figure 5 represents the histogram results obtained separately for the True Positive Rate (TPR = TP/P) and False Positive rate (FPR = FP/N) as a function of the target probability (designed here in logarithmic mode −log_10_(P(C_k_|x)). Figure 5 also shows the difference between both curves, which presents a maximum over P ≈ 10^−4^. At this P value the 78% of true actives will be detected with a false alarm ratio over a 6%. If we increase the threshold, the system reaches higher TPR values at the expenses of increasing the false positives.

The Fig. 5 also represents the behavior of the positive predictive value (PPV = TP/(TP + FP)) and the false discovery rate (FDR = FP/(TP + FP)) for high threshold probability values. A very restrictive system only allowing True Positives could be created by setting the threshold value to P_TH_ = 0.2, but with very poor sensitivity values (1%) (i.e. TPR). In this work we have fixed the decision threshold ‘P_TH_’ to the point in which the PPV and FDR are equal (threshold at 0.004, −log_10_(P(C_k_|x)) = 2.4), thus implying a 74% of Sensitivity.

## Conclusions

In this work we introduced a new physical concept, the molecule’s pairing energies, that can be used to efficiently implement Virtual Screening processes. The model is able to identify active compounds in huge databases by using energy vectors as the basic molecular description. The pairing energy values are dependent on both geometry and the charge distribution inside the molecule that are key factors in the binding process between drugs and targets. When applied to Virtual Screening, the Enrichment Factors obtained are considerably greater than those obtained with other models present in the literature. At the same time, the inclusion of different active compounds to build a single energy-dependent probability distribution function (using the Parzen approximation) further increases the EF and AUC values of the method to values above 50 and 0.8 respectively. Finally we have developed a test evaluation for the activity prediction using the probabilities provided by a Bayesian classifier. The Sensitivity of the system has been analyzed as a function of a probability threshold P_TH_, in order to estimate the expected results. The model can provide reasonable values even when only using two energy parameters (maximum and minimum pairing energies), this fact imply that the memory resources (and therefore the maximum possible volume of the database to be screened) and the processing speed obtained when screening huge molecular databases are considerably enhanced.

## Additional Information

**How to cite this article:** Oliver, A. *et al*. A Bayesian Target Predictor Method based on Molecular Pairing Energies estimation. *Sci. Rep.* **7**, 43738; doi: 10.1038/srep43738 (2017).

**Publisher's note:** Springer Nature remains neutral with regard to jurisdictional claims in published maps and institutional affiliations.

## Supplementary Material

Supplementary Information

## Figures and Tables

**Table 1:** ZINC database codes of most similar compounds retrieved, for 3 different query molecules (which code is in remarked in bold).

Favipiravir	S	Ibuprofen	S	Caffeine	S
**ZINC13915654**	**1.00**	**ZINC38141758**	**1.00**	**ZINC00001084**	1.00
ZINC88190053	0.96	ZINC71767464	1.00	ZINC00000999	1.00
ZINC00330524	0.94	ZINC71767462	1.00	ZINC00004317	1.00
ZINC91873393	0.94	ZINC95080137	1.00	ZINC00036444	1.00
ZINC00337851	0.94	ZINC36157911	1.00	ZINC00039866	1.00
ZINC00337828	0.94	ZINC00002647	1.00	ZINC00040084	1.00
ZINC33961890	0.93	ZINC93141387	1.00	ZINC00040132	1.00
ZINC93874680	0.93	ZINC86364286	1.00	ZINC00040773	1.00
ZINC93892558	0.93	ZINC86364183	1.00	ZINC00041068	1.00
ZINC93892324	0.93	ZINC86350279	1.00	ZINC00047077	1.00

The dimension has been selected to m = 12.

**Table 2:** Average performance of the proposed model compared with different methods present in the literature.

Partial Charges	Model	AUC	STD_AUC_	EF_1%_	STD_EF_
MMFF94	PED (D = 2)	0.646	0.15	17.4	10.1
MMFF94	PED (D = 6)	0.654	0.15	17.4	9.1
MMFF94	PED (D = 12)	0.645	0.15	18.6	9.8
Gasteiger	PED (D = 6)	0.647	0.13	15.1	8.5
—	USR (D = 12)	0.618	0.16	7.9	4.8
Gasteiger	ElectroShape (D = 15)	0.62	0.14	10.8	5.3
MMFF94	ElectroShape (D = 15)	0.67	0.15	13.3	6.0
—	CSR^30^ (D = 12)	0.62	0.11	7.7	4.2
—	MolShacs^22^	0.63	0.08	9.9	2.9

The standard deviation is also shown for an estimation of the variability of AUC and EF. The dimension of the model is also indicated for comparison.

**Table 3:** Results of the number of true positives at the top 5, 10 and 20 in massive VS using DUD + ZINC databases.

Target	Query	Proposed model			USR		
		**TOP 5 (0.25 ppm)**	**TOP 10 (0.5 ppm)**	**TOP 20 (1 ppm)**	**TOP 5 (0.25 ppm)**	**TOP 10 (0.5 ppm)**	**TOP 20 (1 ppm)**
EGFR	ZINC00116727	2	4	8	2	3	3
COX2	ZINC03814636	5	10	16	1	2	2
DHFR	ZINC03814837	0	1	1	0	1	1
EGFR	ZINC03815187	2	2	4	2	2	2
FGFR1	ZINC03815354	0	0	0	0	0	0
PDGFRB	ZINC03815545	0	1	1	1	1	1
HSP90	ZINC03832014	2	5	9	0	0	0
INHA	ZINC03833931	2	2	4	2	2	2
INHA	ZINC03833949	0	0	4	0	0	0
VEGFR2	ZINC03834201	0	0	0	0	0	0

The 3rd, 4th and 5th columns are the results obtained with the proposed descriptors. The 6th, 7th and 8th columns are the results with the USR model. Both models present the same screening speed since they are using the same number of descriptors (m = 12).
