# Supplementary material for: A Bayesian Target Predictor Method based on Molecular Pairing Energies estimation
Source: Sci Rep. 2017 Mar 6;7:43738. doi: 10.1038/srep43738 (PMC5338323; doi:10.1038/srep43738)
Supplement: Supplementary Information [file srep43738-s1.pdf]

Supplementary Information

A Bayesian Target Predictor Method based on Molecular Pairing Energies estimation

Antoni Oliver*,[1], Vincent Canals[1], and Josep L. Rosselló*,[1]

[1]Physics Department. Universitat de les Illes Balears. Correspondence and requests for materials should be addressed to J.L Rosselló and A. Oliver (email: j.rossello@uib.es and a.oliver@uib.es)

We present the figures of the top 10 most similar molecules found using ZINC All purchasable compounds. This set has been processed with the proposed descriptors, PCE using expression (3) as a similarity metric for comparisons. The query molecules are Favipiravir, Ibuprofen and Caffeine. The molecules has been first filtered using a similarity threshold value (0.9) and ranked by similarity score. Finally, the first 10 molecules have shown for each query.

Favipiravir Query

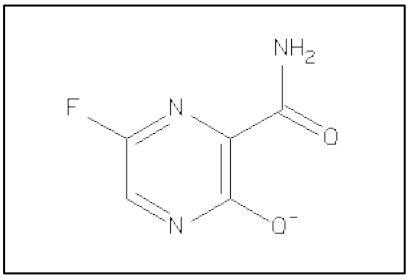

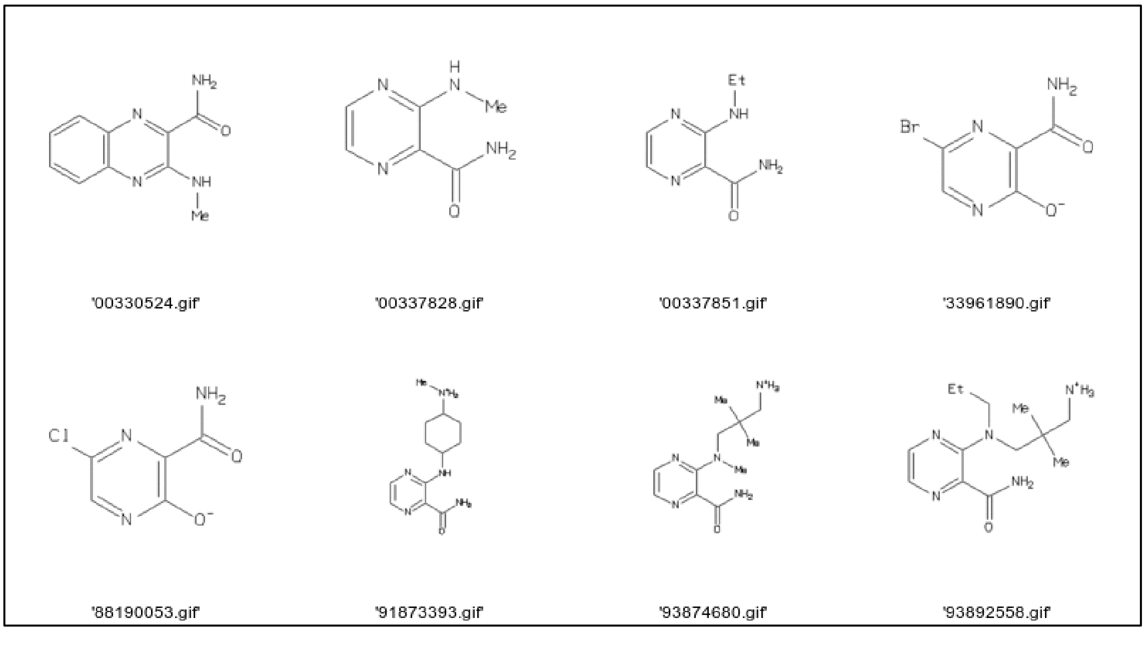

Ibuprofen Query

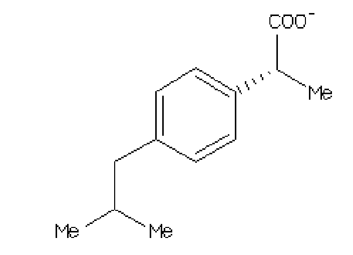

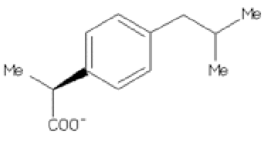

'00002647.gif'

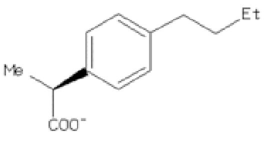

'36157911.gif'

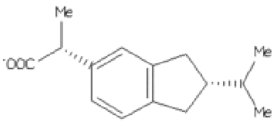

'71767462.gif'

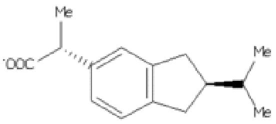

'71767464.gif'

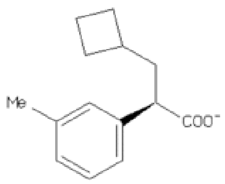

'86364183.gif'

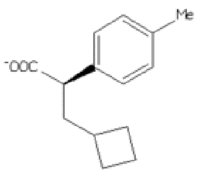

'86364286.gif'

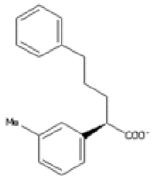

'93141387.gif'

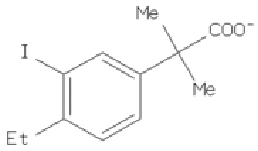

'95080137.gif'

Caffeine Query

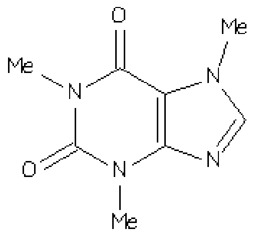

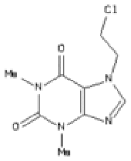

'00000999.gif'

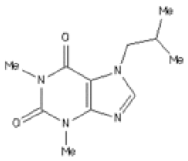

'00004317.gif'

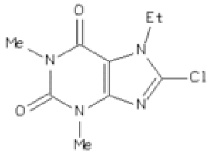

'00036444.gif'

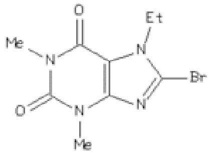

'00039866.gif'

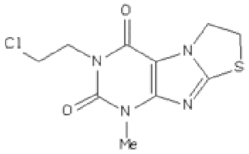

'00040084.gif'

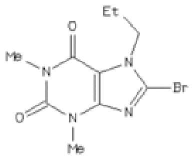

'00040132.gif'

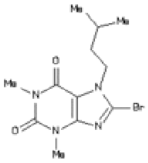

'00040773.gif'

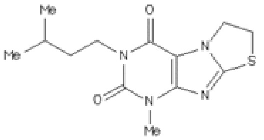

'00041068.gif'
